# Reduced expression of PinX1 correlates to progressive features in patients with prostate cancer

**DOI:** 10.1186/1475-2867-14-46

**Published:** 2014-06-06

**Authors:** Rong Shi, Zhen Zhao, Hui Zhou, Min Wei, Wen-Li Ma, Jue-Yu Zhou, Wan-Long Tan

**Affiliations:** 1 Institute of Genetic Engineering, Southern Medical University, Guangzhou 510515, China; 2 Department of Urinary Surgery, Nanfang Hosptial, Southern Medical University, Guangzhou 510515, China

**Keywords:** PinX1, Prostate cancer, Tissue microarray, Immunohistochemistry

## Abstract

**Background:**

Pin2/TRF1 binding protein X1 (PinX1) has been identified as an endogenous telomerase inhibitor and a major haploinsufficient tumor suppressor gene. Increasing evidence suggests that reduced expression of PinX1 plays a key role in tumorigenesis. However, the PinX1 expression status and its correlation with the clinicopathological features in prostate cancer (PCa) have not been investigated.

**Methods:**

PinX1 mRNA and protein expression in PCa and adjacent normal prostate tissues were evaluated by real-time quantitative RT-PCR (qRT-PCR) and western blotting. The clinicopathological significance of PinX1 was investigated by immunohistochemistry (IHC) analysis on a PCa tissue microarray (TMA). The cut-off score for positive expression of PinX1 was determined by the receiver operating characteristic (ROC) analysis. The correlation between PinX1 expression and clinicopathological features of PCa was analyzed by Chi-square test.

**Results:**

Reduced expression of PinX1 mRNA and protein was observed in the majority of PCa, compared with their paired adjacent normal prostate tissues. When PinX1 positive expression percentage was determined to be above 60% (area under ROC curve = 0.833, P = 0.000), positive expression of PinX1 was observed in 100% (8/8) of normal prostate tissues and 32.5% (13/40) of PCa tissues by IHC. Reduced expression of PinX1 in patients was correlated with advanced clinical stage (χ^2^ = 10.230, p = 0.017), high Gleason score (χ^2^ = 4.019, p = 0.045), positive regional lymph node metastasis (χ^2^ = 10.852, p = 0.004) and distant metastasis (χ^2^ = 7.965, p = 0.005).

**Conclusions:**

Our findings suggest that reduced expression of PinX1 is correlates to progressive features in patients with PCa and may serve as a potential marker for diagnosis.

## Background

Prostate cancer (PCa) is the second most frequently diagnosed cancer and the sixth leading cause of cancer death in males worldwide [1,2]. The most common histological subtype of PCa is adenocarcinoma, which derives from the semen-secreting prostate gland cells. Although most PCa present as noninvasive adenocarcinoma, there are cases of aggressive PCa which could metastasize primarily to the bones [3,4], and also lymph nodes, rectum, bladder, lower ureters, etc. To date, the most important clinical prognostic indicators of PCa are pTNM stage, Gleason score and pre-therapy PSA level. However, the mechanism contributes to the progression of PCa remains unclear and many variations among individual patients are unexplained. Thus, novel biomarkers are strongly needed to enable more accurate diagnosis, prognosis and individualized medication of PCa [5].

Pin2/TRF1 binding protein X1 (PinX1) has been identified as an endogenous telomerase inhibitor and a major haploinsufficient tumor suppressor gene localized at human chromosome 8p23, a region with frequent loss of heterozygosity in a number of human cancers [6]. Increasing evidence suggest that reduced expression of PinX1 plays a key role in tumorigenesis. Zhou et al. reported that PinX1 expression was reduced in most human breast cancer tissues and cell lines, PinX1 allele loss caused majority of mice to develop a variety of epithelial cancers, the mechanism involves chromosome instability which recapitulated to 8p23 allele loss in humans [7,8]. Cai et al. suggested that decreased expression of PinX1 was correlated with poor prognostic factors of ovarian carcinoma and loss of PinX1 was an adverse independent molecular marker for epithelial ovarian carcinoma patients [9]. Kondo et al. reported that LOH of PinX1 locus and hypoacetylation of histone H4 in the 5' UTR of PinX1 were associated with reduced expression of PinX1 in gastric carcinoma [10]. Ma et al. reported that LOH of PinX1 was associated with the TNM stage of the gastric carcinoma specimens, and could be regarded as a sign of gastric cancer development [11]. Park et al. suggested that LOH of PinX1 might occur as an early event in the development of hepatocellular carcinoma, especially in the cases with HBV infection [12]. Moreover, the role of PinX1 as a putative tumor suppressor was proved by several other groups in different cancer cell lines [13-18]. However, the PinX1 expression status and its correlation with the clinicopathological features in PCa have not been investigated.

In this study, PinX1 mRNA and protein expression in PCa and adjacent normal prostate tissues were evaluated by real-time quantitative RT-PCR (qRT-PCR) and western blotting. Immunohistochemistry (IHC) analysis for PinX1 was performed on a PCa tissue microarray (TMA). Here we report the expression of PinX1 mRNA and protein was decreased in PCa. Reduced expression of PinX1 was correlated to progressive features in patients with PCa.

## Results

### Expression of PinX1 mRNA and protein in paired PCa and adjacent normal prostate tissues

**Figure 1:**
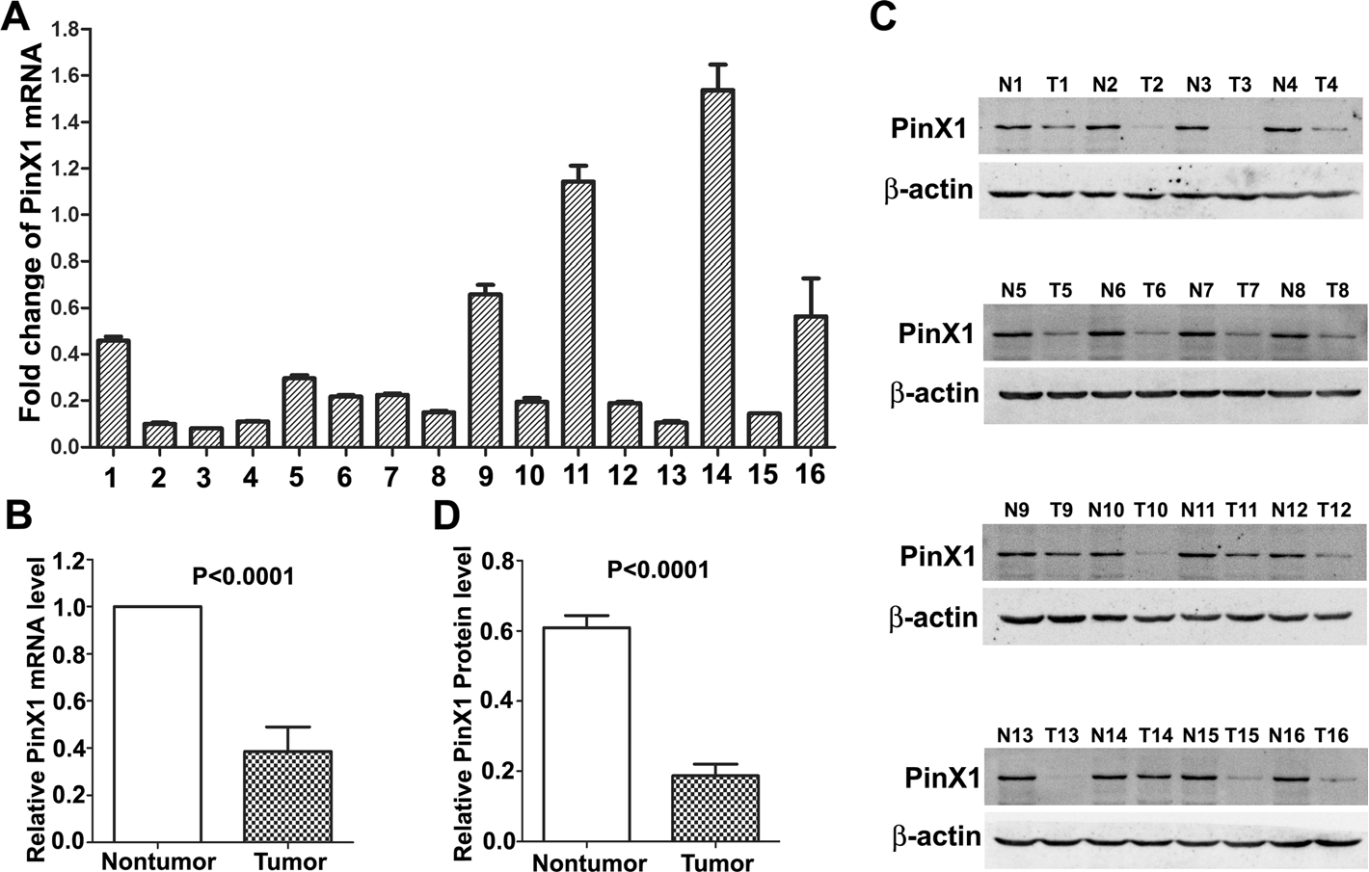
**qRT-PCR and Western blot analysis of PinX1 expression in paired PCa and adjacent normal prostate tissues. (A)** Fold changes (2^-△△Ct^ values) by qRT-PCR showed a reduced expression of PinX1 mRNA in the majority of PCa cases (14/16),when compared with paired normal prostate tissues. Expression levels were normalized for GAPDH. **(B)** Significant differences of PinX1 mRNA expression between the PCa and adjacent normal prostate tissues (P < 0.0001). **(C)** Western blotting indicated down-regulation of PinX1 protein in PCa tissues (15/16) in comparison with the adjacent normal prostate tissues. β-actin was used as internal control. T, PCa; N, normal. **(D)** Significant differences of PinX1 protein expression between the PCa and adjacent normal prostate tissues (P < 0.0001).

The qRT-PCR result showed that in 14 of the 16 sample pairs, fold changes (the 2^-△△Ct^ values) were less than 1 between PCa and adjacent normal prostate tissue (Figure 1A), which indicated the PinX1 mRNA expression was down-regulated in PCa tissues compared to the adjacent normal prostate tissues. The mean fold change is 0.385 and paired t-test showed that difference between the two groups was statistically significant (P < 0.0001, Figure 1B). Western blotting analysis also demonstrated down-regulation of the PinX1 protein in 15 of the 16 PCa tissues compared to their adjacent normal counterparts (Figure 1C). The mean optical density value of the PCa tissues is 0.306 fold of the adjacent normal tissues. The difference of the PinX1 protein expression between the two groups was also statistically significant by paired t-test (P < 0.0001, Figure 1D).

### Expression of PinX1 in PCa by IHC and cut-off score selection

**Figure 2:**
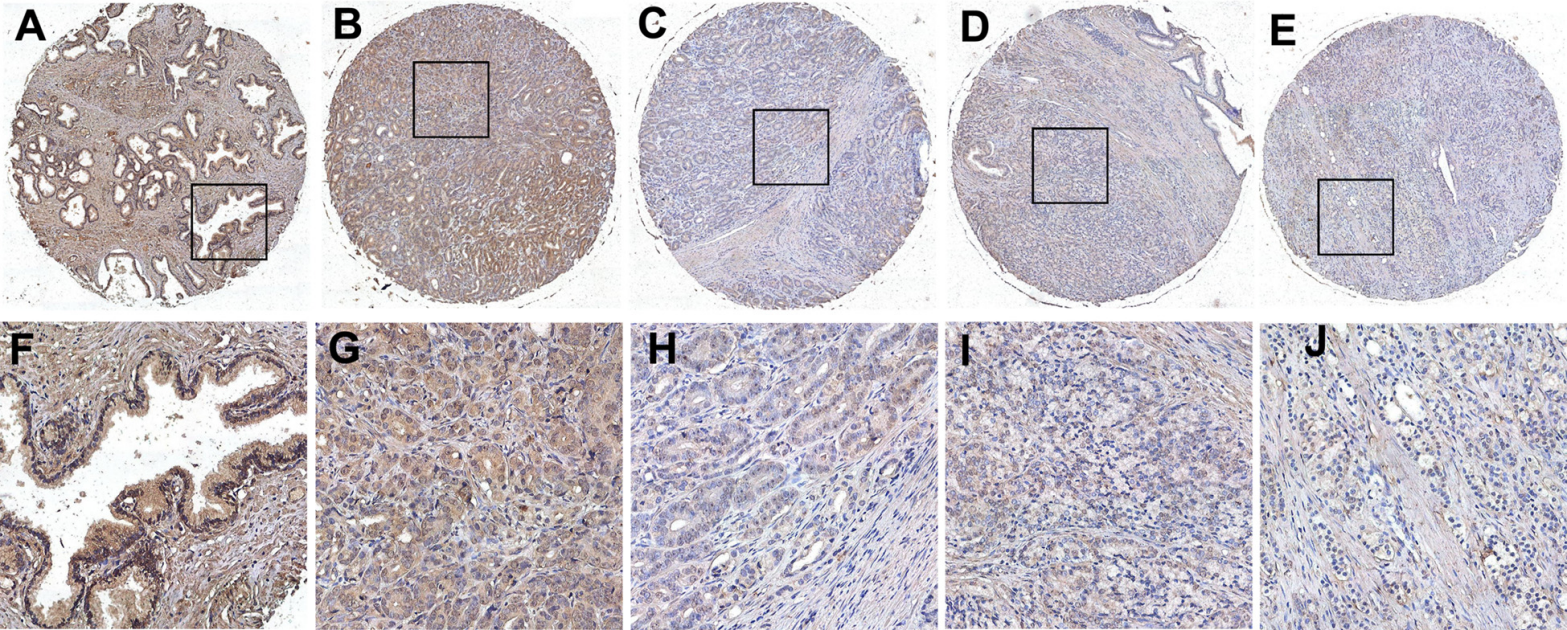
**The expression of PinX1 protein in normal prostate and PCa tissues by IHC on the TMA. (A)** Positive expression of PinX1 in normal prostate tissue, in which about 100% of the prostate epithelial cells demonstrated a strong nuclear staining of PinX1, while a lesser staining in the cytoplasm could also be observed (100x). **(B)** Positive expression of PinX1 in PCa (case 21), in which more than 90% of tumor cells showed positive staining (100x). **(C)** Negative expression of PinX1 in PCa (case 11), with less than 40% positive tumor cells (100x). **(D)** Negative expression of PinX1 in PCa (case 27), with less than 35% positive tumor cells (100x). **(E)** Negative expression of PinX1 in PCa (case 25), with less than 15% positive tumor cells (100x). **(F)**, **(G)**, **(H)**, **(I)** and **(J)** demonstrate the higher magnification (400x) from the area of black square in **(A)**, **(B)**, **(C)**, **(D)** and **(E)**, respectively.

**Figure 3:**
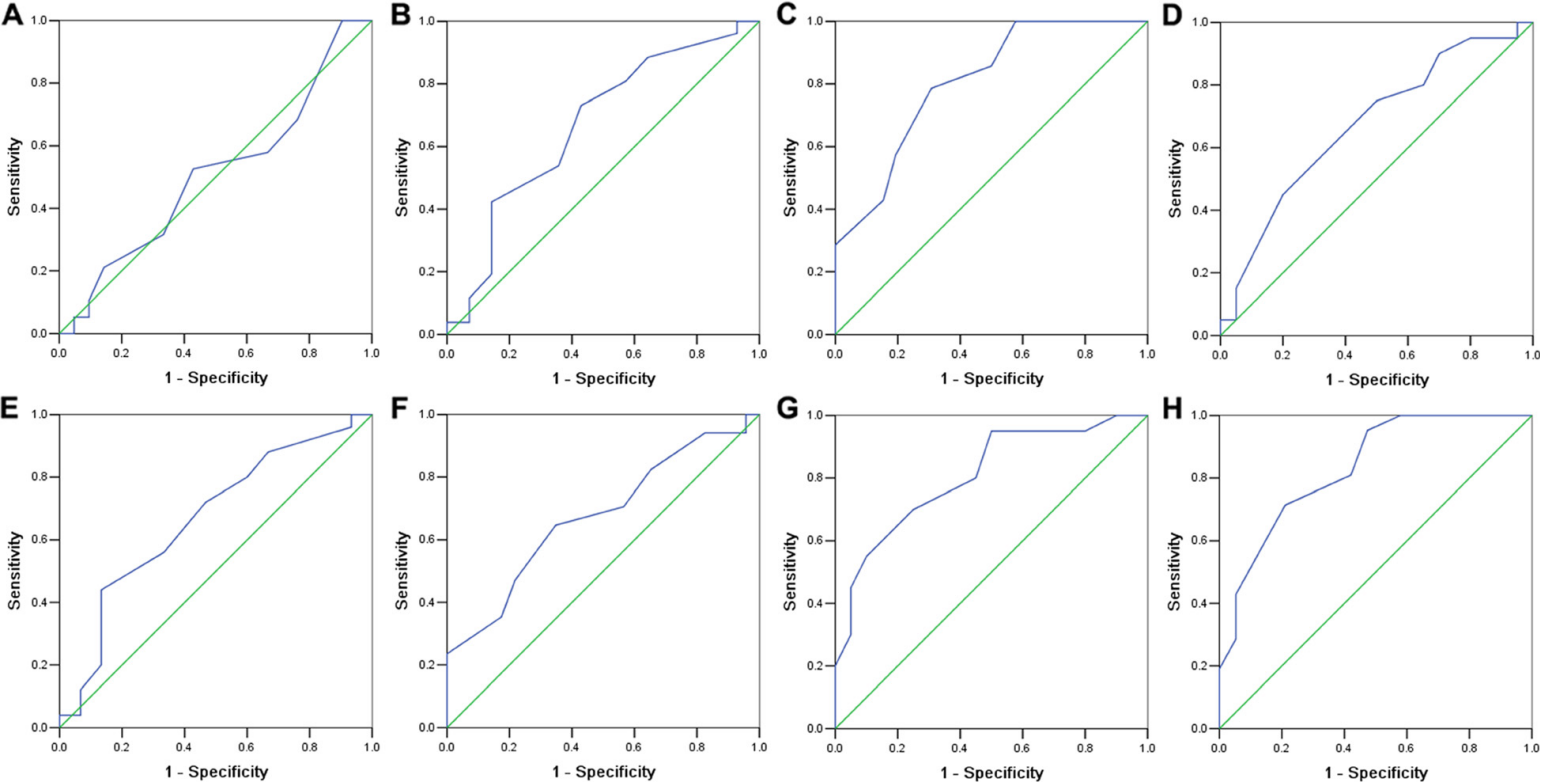
**Selection of the optimum cut-off score for positive expression of PinX1 by receiver operating characteristic (ROC) analysis.** Various ROC curves were plotted by sensitivity and specificity for each outcome: age **(A)**, histological grade **(B)**, clinical stage **(C)**, Gleason pattern **(D)**, Gleason grade **(E)**, pT status **(F)**, pN status **(G)**, pM status **(H)**.

The subcellular localization and expression of the PinX1 protein were observed and scored by IHC on the TMA, which including 40 prostate adenocarcinoma and 8 normal prostate tissues. IHC result showed that the PinX1 protein was primarily stained in cell nucleus, while in the cytoplasm, a lesser staining could also be observed (Figure 2). The receiver operating characteristic (ROC) curve for all the clinicopathological features at different PinX1 scope were plotted (Figure 3), the corresponding area under the ROC curve (AUC) and P-value were analyzed by the SPSS 13.0 software (Table 1). Optimal cut-off score for PinX1 was determined by the ROC curve for pM which showed the shortest distance to the point (0.0,1.0) and could maximize both the sensitivity and specificity. Based on the cut-off score, tissues were defined as PinX1 positive when PinX1 expression percentage was above 60%. According to the PinX1 scores for each sample and the cut-off score determined by the ROC curve analysis, positive expression of PinX1 was detected in 32.5% (13/40) of PCa and 100% (8/8) of the normal prostate tissues.

**Table 1:** Area under the ROC curve for each clinicopathological features

**Characteristics**	**AUC (95% CI)**	**P-value**
Age	0.508 (0.352-0.690)	0.935
Grade	0.668 (0.485-0.850)	0.084
Stage	0.805 (0.670-0.940)	0.002
Gleason pattern	0.671 (0.503-0.840)	0.064
Gleason score	0.668 (0.491-0.845)	0.078
pT	0.675 (0.503-0.848)	0.061
pN	0.808 (0.673-0.942)	0.001
pM	0.833 (0.709-0.957)	0.000

ROC, the receiver–operator curve; AUC, area under the curve.

### Relationship between PinX1 expression and clinicopathological characteristics of PCa patients

The relationship between PinX1 scores with respect to patient clinicopathological features was listed in Table 2. The result indicated that reduced expression of PinX1 in patients was correlated with advanced clinical stage (χ^2^ = 10.230, p = 0.017), high Gleason score (χ^2^ = 4.019, p = 0.045), positive regional lymph node metastasis (χ^2^ = 10.852, p = 0.004) and distant metastasis (χ^2^ = 7.965, p = 0.005). There was no statistically significant correlations between PinX1 expression and other features, such as age,histology grade, Gleason pattern or pT status (P > 0.05).

**Table 2:** Association of PinX1 expression with clinicopathological characteristics of PCa patients

**Characteristics**	**PinX1 staining**		**Total**	**Pearson Chi-square**	**P value**^**b**^
	**Negative (%)**	**Positive (%)**			
Age at surgery (years)					
≤67^a^	13 (72.2)	5 (27.8)	18	0.333	0.564
>67	14 (63.6)	8 (36.4)	22		
Histology grade					
I	4 (50.0)	4 (50.0)	8	3.569	0.168
II	11 (61.1)	7 (38.9)	18		
III	12 (85.7)	2 (14.3)	14		
Clinical stage					
I	0 (0.0)	1 (100.0)	1	10.230	0.017
II	6 (46.2)	7 (53.8)	13		
III	3 (50.0)	3 (50.0)	6		
IV	18 (90.0)	2 (10.0)	20		
Gleason pattern					
1	1 (33.3)	2 (66.7)	3	4.177	0.383
2	6 (66.7)	3 (33.3)	9		
3	4 (50.0)	4 (50.0)	8		
4	9 (81.8)	2 (18.2)	11		
5	7 (77.8)	2 (22.2)	9		
Gleason score					
1-7	14 (56.0)	11 (44.0)	25	4.019	0.045
8-10	13 (86.7)	2 (13.3)	15		
pT status					
T1	0 (0.0)	1 (100.0)	1	7.322	0.062
T2	9 (52.9)	8 (47.1)	17		
T3	11 (73.3)	4 (26.7)	15		
T4	7 (100.0)	0 (0.0)	7		
pN status					
N0	9 (45.0)	11 (55.0)	20	10.852	0.004
N1	17 (94.4)	1 (5.6)	18		
N2	1 (50.0)	1 (50.0)	2		
pM status					
M0	10 (47.6)	11 (52.4)	21	7.965	0.005
M1	17 (89.5)	2 (10.5)	19		

^a^Median age.

^b^P value are from Chi-square test.

## Discussion

At prescent, PSA is still the most commonly used molecular markers for the diagnosis of PCa. However, due to the drawback of PSA as a nonspecific biomarker, nontumor patients who underwent diagnostic prostate biopsies and clinically insignificant PCa patients who received overtreatments had increased globally. This had not only placed great physical and psychological burden on the patients, but also increased the cost on health care of the governments. Therefore, novel biomarkers such as PCA3, TMPRSS2-ERG and some microRNAs [19] etc. have been assessed for their diagnostic and prognostic role in PCa, especially the detection of these biomarker in the body fluid such as blood, urine or seminal fluid.

Tissue biomarkers such as Ki-67, PTEN, E-Cadherin, EZH2, SPINK and ERG are important for evaluating the prognosis of the patients after the prostate biopsies or radical prostatectomy. Khatami et al. [20]. proved the correlation of Ki-67 to prostate-specific antigen doubling time (PSADT), Gleason score, and its role as a valuable prognostic marker of PSA relapse after radical prostatectomy in men with screen-detected, low-grade, low-stage PCa. Ritu et al. [21] developed a novel dual color immunohistochemical method recently which could simultaneously detect the ERG-PTEN and ERG-SPINK1 status in PCa. In addition, novel gene panel which detect and score the expression of 31 genes simultaneously was also developed for evaluating the prognosis of PCa [22,23]. Although the progress appears to be encouraging, there is still an urgent need for new objective strategies and novel biomarkers for diagnosis, prognosis and evaluation of drug resistance.

PinX1 is a newly identified telomere-associated protein which was recruited to the telomeres by TRF1, provided a critical link between TRF1 and telomerase inhibition to help maintain telomere homeostasis [24]. Reduced expression of PinX1 has been implicated in various human cancers, such as breast cancer [7], ovarian cancer [9], gastric cancer [10,11] and liver cancer [12], which suggested a tumor suppressor role of pinX1 in multiple human cancers. However, the relationship between PinX1 expression status and clinicopathological features of PCa has not been reported so far. In this study, qRT-PCR and western blotting were applied to examine the expression levels of both the PinX1 mRNA and protein in paired PCa and adjacent normal prostate tissue samples. The results were in line with expectations that reduced expression of PinX1 mRNA and protein were observed in the majority of PCa, when compared with their paired adjacent normal prostate tissues (P < 0.05).

In addition, the expression of PinX1 protein was examined by IHC on a TMA including 40 adenocarcinoma and 8 normal prostate tissues. Our results demonstrated that reduced expression of PinX1 was frequently observed in PCa tissues, whereas all the normal prostate tissues showed a deep staining of PinX1. This was in accord with the qRT-PCR and western blotting results. Further correlation analysis revealed that reduced expression of PinX1 was correlated to higher Gleason score and poor prognosis regarding presence of advanced clinical stage, higher N status and higher M status. These findings suggest that, as a major haploinsufficient tumor suppressor gene, PinX1 expression was correlated to the risk of PCa progression and may serve as a potential marker for the diagnosis of PCa patients. Further investigation for the mechanisms of PinX1 in PCa carcinogenesis and validation in large clinical trials are necessary for subsequently implement of PinX1 as a biomarker in the clinical practice.

## Conclusions

Our findings suggest that PinX1 was down-regulated in PCa compared to adjacent normal prostate tissues. Reduced expression of PinX1 is significantly correlated with low differentiation and adverse clinical features in PCa. PinX1 may serve as a potential marker for the diagnosis of PCa.

## Methods

### Patients and primary PCa samples

In this study, 16 fresh paired prostate adenocarcinoma and adjacent normal prostate tissue samples were collected from patients for qRT-PCR and western blotting analysis between September 2011 and June 2013. A total of 48 paraffin-embedded tissues diagnosed between 2003 and 2008 was retrieved for TMA construction and IHC analysis. All the samples were collected from the Department of Urinary Surgery, Nanfang Hospital of Southern Medical University (Guangzhou, China). The samples selected were pathologically diagnosed cases of PCa, having received no prior chemotherapy or radiotherapy before surgery. Ages of the 40 patients with PCa ranged from 22 to 78 years (median, 67 years), clinicopathological features of patients including age at diagnosis, histological grade, Gleason pattern, Gleason score, clinical stage and pTNM status were listed in Additional file 1: Table S1. Written informed consent was obtained from all patients for use of the tissue samples and clinical records. The study protocol was performed under the approval by the Ethic Committee of the Nanfang Hospital.

### RNA isolation and qRT-PCR analysis

Total RNAs were isolated from 16 pairs of fresh PCa and adjacent normal prostate tissues using RNAiso Plus reagent (Takara, Dalian, China). RNA concentration and OD_260/280_ were assessed by using a Biophotometer plus spectrophotometer (Eppendorf, Germany). The integrity of total RNAs were confirmed by denaturing agarose gel electrophoresis. Qualified total RNAs were reversely transcribed into first-strand cDNAs by using the PrimeScript® RT reagent Kit (Takara, Dalian, China). The 50 μL PCR reaction included 80 ng of the cDNA template, 0.4 μM of the forward and reverse primers each, 25 μL of the 2× SYBR Premix Ex Taq™ II (Takara, Dalian, China), and ddH_2_O. For the PinX1 gene, the forward primer was 5’- CCAGAGGAGAACGAAACCACG -3’, and the reverse primer was 5’- ACCTGCGTCTCA GAAATGTCA -3’. For the GAPDH gene, the forward primer was 5’- CTGGGCTACACTGAGCACC -3’, and the reverse primer was 5’- AAGTGGTCGTTGAGGGCAA TG -3’. The PCR was performed in an ABI 7500 real-time PCR amplifier (ABI, USA) with an initial denaturing temperature of 95°C for 30 s, followed by 40 cycles of 95°C for 5 s, 60°C for 34 s. The Ct values were acquired using the 7500 system SDS software (ABI, USA) with manual thresholds. Expression of PinX1 was normalized by using GADPH as an internal control. The fold changes between PCa and normal tissue pairs were analyzed by calculating the 2^-△△Ct^ values. Each PCR reaction was repeated in triplicate for stable results.

### Western blotting analysis

Total proteins from 16 pairs of fresh PCa and adjacent normal tissues were extracted using radio-immunoprecipitation assay (RIPA) buffer containing 1 mM PMSF (Beyotime, Haimen, China). Then protein concentration was determined by the TECAN Infinite 200 microplate reader (TECAN, Austria) at 562 nm using BCA Protein Assay Kit (Beyotime, Haimen, China). 50 μg of each protein sample was loaded on the 10% SDS polyacrylamide gels for electrophoresis. Proteins separated on the gel were then transferred to polyvinylidene fluoride (PVDF) membrane (Millipore, USA) by a Transblot SD Cell semi-dry transfer machine (BioRad, USA). The membranes were blocked by 5% nonfat milk. Goat polyclonal anti-PinX1 antibody (1:500, Santa Cruz Biotechnology, Santa Cruz, USA) and HRP conjugated rabbit anti-goat IgG (1:5000, Multisciences, Hangzhou, China) were used to detect the PinX1 protein. Mouse monoclonal anti-β-actin antibody (1:2000, Multisciences, Hangzhou, China) and HRP conjugated goat anti-mouse IgG antibody (1:5000, Multisciences, Hangzhou, China) were used to detect β-actin. BeyoECL Plus Kit (Beyotime, Haimen, China) was used for the detection of HRP on the antigen-antibody complex. Finally, the result of western blotting was visualized by the Image Station 4000R PRO scanner (CareStream Health, U.S.A.).

### TMA construction and IHC

Representative sections of PCa or normal prostate in the pre-existing paraffin-embedded tissue blocks were determined according to the overlaid H&E staining slides. The TMA was done by using a needle to punch a 1.8 mm diameter cylinder in the representative section of each block, and placing the cylinders into an array on a recipient paraffin block. 5 μm thick multiple sections were cut from the TMA block and mounted on microscope slides for immuohistochemistry analysis. The TMA slides were dried overnight at 37°C, deparaffinized in xylene and then rehydrated in graded ethanol. The endogenous hydrogen peroxidase activities of the TMA were inhibited by immersing the slide into 3% H_2_O_2_. Antigen retrieval was done by microwave heating with sodium citrate buffer (pH 6.0) at 100°C for 20 min. The TMA slides were blocked with 5% normal goat serum at room temperature for 30 min, followed by incubation with polyclonal anti-PinX1 antibody (1:200, Santa Cruz Biotechnology, Santa Cruz, USA) at 4°C overnight, and polymer peroxidase-labeled secondary antibody (ZSGB-Bio, Beijing, China) at room temperature for 1 h. The TMA slides were stained by using the DAB Horseradish Peroxidase Color Development Kit (Beyotime, Haimen, China), and then counterstained by hematoxylin. Phosphate buffered saline replaced anti-PinX1 primary antibody was used as a negative control, and known IHC positive slide was used as a positive control.

### IHC evaluation

Two investigators graded the immunohistochemical expression of PinX1 independently using a semi-quantitative scoring method according to distribution, intensity, and percentage of positive cells. With regard to distribution of PinX1 protein, the scores were expressed as percentage of positive tumor cells over the total tumor cells, by using 5% increments (0, 5%, 10%…100%). The scores were accepted if both of the two investigators agree with the values. Otherwise, the values were re-estimated until a consensus was reached.

### Selection of cut-off score and statistical analysis

Receiver operating characteristic (ROC) curve analysis was applied for acquiring an optimal cut-off score for positive expression of PinX1 using the 0,1-criterion. The ROC curves were generated by plotting the sensitivity and specificity for each outcome at different PinX1 scores. The score which was closest to the point [0.0, 1.0] on the curve with both maximum sensitivity and specificity was selected as the cut-off value. Dichotomization of the clinicopathological features for ROC analysis were as follow: age (≤67 vs. >67),histology grade (I-II vs. III), Gleason pattern ( 1–3 vs. 4–5 ), Gleason pattern (2–7 vs. 8–10), clinical stage(I-II vs.III-IV), pT stage(T1-T2 vs.T3-T4), pN stage(N0 vs. N1-N2) and pM stage(M0 vs. M1). Statistical analyses were performed by SPSS 13.0 software (SPSS, Chicago, IL, USA). The association of PinX1 expression with clinicopathological features of PCa patients was analyzed by the Chi-square test. Differences were considered significant when the P-value was <0.05 (two-tailed test ).

## Competing interests

The authors declare that they have no competing interests.

## Authors’ contributions

RS and ZZ carried out the molecular, pathological and immunological experiments and drafted the manuscript. WLT, JYZ and WLM participated in the design of the study and coordination. RS, WM and HZ performed the statistical analysis. All authors read and approved the final manuscript.

## Supplementary Material

Additional file 1: Table S1 Baseline demographic characteristics of the patients with PCa for constructing the TMA and the IHC results of PinX1. Click here for file
