# Supplementary material for: Reduced expression of PinX1 correlates to progressive features in patients with prostate cancer
Source: Cancer Cell Int. 2014 Jun 6;14:46. doi: 10.1186/1475-2867-14-46 (PMC4059453; doi:10.1186/1475-2867-14-46)
Supplement: Additional file 1: Table S1 — Baseline demographic characteristics of the patients with PCa for constructing the TMA and the IHC results of PinX1. [file 1475-2867-14-46-S1.doc]

Table S1. Baseline demographic characteristics of the patients with PCa for constructing the TMA and the IHC results of PinX1

| Age | Grade | Stage | Gleason Pattern | Gleason Score | pT | pN | pM | PinX1 IHC |
| --- | --- | --- | --- | --- | --- | --- | --- | --- |
| 22 | I | III | 2 | 5 | 3 | 0 | 0 | + |
| 51 | II | II | 4 | 6 | 2 | 0 | 0 | + |
| 56 | II | II | 4 | 9 | 2 | 0 | 0 | - |
| 60 | I | IV | 1 | 2 | 4 | 1 | 1 | - |
| 60 | II | IV | 4 | 7 | 3 | 1 | 1 | - |
| 60 | II | IV | 3 | 7 | 3 | 1 | 0 | - |
| 61 | I | III | 2 | 5 | 3 | 1 | 0 | - |
| 62 | II | IV | 4 | 7 | 3 | 1 | 1 | - |
| 62 | III | II | 4 | 9 | 2 | 0 | 0 | - |
| 63 | III | IV | 5 | 10 | 2 | 1 | 1 | - |
| 64 | I | I | 1 | 3 | 1 | 0 | 0 | + |
| 64 | III | IV | 4 | 8 | 3 | 0 | 1 | - |
| 64 | III | II | 5 | 10 | 2 | 0 | 0 | + |
| 65 | I | II | 2 | 3 | 2 | 0 | 0 | + |
| 65 | III | IV | 5 | 10 | 2 | 1 | 1 | - |
| 66 | I | IV | 2 | 3 | 3 | 1 | 1 | - |
| 66 | III | II | 4 | 9 | 2 | 0 | 0 | - |
| 67 | III | II | 5 | 10 | 2 | 0 | 0 | - |
| 68 | II | II | 3 | 7 | 2 | 0 | 0 | + |
| 69 | II | III | 2 | 5 | 3 | 0 | 0 | + |
| 69 | II | II | 2 | 6 | 2 | 0 | 0 | - |
| 70 | II | III | 3 | 7 | 3 | 0 | 0 | + |
| 70 | III | IV | 5 | 10 | 4 | 1 | 1 | - |
| 71 | II | II | 5 | 9 | 2 | 0 | 0 | - |
| 71 | III | III | 4 | 9 | 3 | 0 | 0 | - |
| 72 | I | II | 1 | 3 | 2 | 0 | 0 | + |
| 72 | II | II | 3 | 7 | 2 | 0 | 0 | + |
| 73 | II | IV | 2 | 6 | 3 | 1 | 1 | - |
| 73 | II | II | 3 | 5 | 2 | 0 | 0 | + |
| 73 | II | III | 4 | 6 | 3 | 0 | 0 | - |
| 73 | II | IV | 4 | 7 | 3 | 1 | 1 | + |
| 73 | III | IV | 5 | 10 | 4 | 1 | 1 | - |
| 74 | II | IV | 2 | 5 | 4 | 1 | 1 | - |
| 75 | I | IV | 2 | 3 | 2 | 1 | 1 | - |
| 75 | II | IV | 3 | 7 | 4 | 1 | 1 | - |
| 75 | III | IV | 3 | 7 | 4 | 1 | 1 | - |
| 76 | III | IV | 5 | 10 | 3 | 1 | 1 | - |
| 77 | III | IV | 5 | 10 | 2 | 2 | 1 | + |
| 78 | II | IV | 3 | 5 | 3 | 2 | 1 | - |
| 78 | III | IV | 4 | 9 | 4 | 1 | 1 | - |
